# Exploring the characteristics of newly defined at-risk drinkers following the change to the UK low risk drinking guidelines: a retrospective analysis using Health Survey for England data

**DOI:** 10.1186/s12889-019-7240-0

**Published:** 2019-07-08

**Authors:** Philippa Case, Linda Ng Fat, Nicola Shelton

**Affiliations:** Department of Epidemiology and Public Health, UCL 1-19 Torrington Place, London, WC1E 7HB UK

**Keywords:** Alcohol, Drinking guidelines, Health survey for England, Alcohol risk, Sociodemographic characteristics, Alcohol screening

## Abstract

**Background:**

Alcohol guidelines enable individuals to make informed choices about drinking and assist healthcare practitioners to identify and treat at-risk drinkers. The UK *Low Risk Drinking Guidelines* were revised in 2016 and the weekly guideline for men was reduced from 21 to 14 units per week. This study sought to retrospectively establish 1) the number of additional at-risk male drinkers in England, 2) which demographic characteristics were associated with being an at-risk drinker under the previous versus new guidelines.

**Methods:**

Average weekly alcohol consumption for men aged 16+ from the cross-sectional nationally representative Health Survey for England were used to 1) calculate annual population prevalence estimates for newly defined at-risk (> 14 to ≤21 units/week) male drinkers from 2011 to 2015 (*N* = 3487–3790), and 2) conduct logistic regression analyses for at-risk vs low risk male drinkers under the previous (> 21 vs ≤21 units/week) and new (> 14 vs ≤14 units/week) guidelines to assess characteristics associated with being at-risk drinkers under each guideline using 2015 data (*N* = 2982).

**Results:**

Population prevalence estimates of newly defined at-risk drinkers ranged from 10.2% (2014 = 2,182,401 men)-11.2% (2011 = 2,322,896 men). Under the new guidelines, men aged 55–74 (OR = 1.63,95% CI = 1.25–2.12); men in managerial/professional occupations (OR = 1.64,95% CI = 1.34–2.00); current smokers (OR = 2.26,95% CI = 1.73–2.94), ex-regular smokers (OR = 2.01,95% CI = 1.63–2.47) and ex-occasional smokers (OR = 1.85,95% CI = 1.25–2.74); men from the North East (OR = 2.08,95% CI = 1.38–3.13) and North West (OR = 1.91,95% CI = 1.41–2.60) of England all had greater odds, and non-white men had reduced odds (OR = 0.53,95% CI = 0.34–0.80) of being at-risk drinkers, as they had under the previous guidelines. Under the new guidelines only: a higher percentage of at-risk drinkers aged 16–34 (32% vs 19%) attenuated the odds of men aged 35–54 being at-risk (OR = 1.18,95% CI = 0.92–1.51); a higher percentage of married at-risk drinkers (37% vs 24%) attenuated the odds of single men being at-risk (OR = 1.28,95% CI = 0.99–1.67); men from the West Midlands (OR = 1.68,95% CI = 1.17–2.42) and London (OR = 1.53,95% CI = 1.03–2.28) had greater odds of being at-risk drinkers.

**Conclusions:**

The change to the *Low Risk Drinking Guidelines* would have resulted in more than 2 million additional male at-risk drinkers in England. Most groups with greater odds of being at-risk drinkers under the new guidelines were those already known to be drinking the most, strengthening the case for targeted screening and education. Additionally, under the new guidelines, a marked proportion of 16–35 year olds and married men were at-risk and men in the West Midlands and London had greater odds of being at-risk drinkers. These groups may benefit from specific education around the new *Low Risk Drinking Guidelines*.

## Background

In England, alcohol guidelines form part of a suite of population level interventions to address problematic alcohol consumption [1, 2]. They are used to aid individuals and healthcare professionals to assess the health risks associated with alcohol consumption [3, 4]. The UK *Low Risk Drinking Guidelines* were changed in 2016, reducing the maximum weekly alcohol consumption guideline down from 21 to 14 units per week for men, due to new evidence of the health risks associated with drinking at lower levels [3]. To assist with targeted screening for alcohol risk in healthcare services and targeted alcohol education programmes, this study used annual, nationally representative survey data to retrospectively explore how many men would have been redefined from low risk to at-risk drinkers according to the new guidelines, which demographic characteristics were associated with being an at-risk drinker before and after the change in guidelines and whether the characteristics associated with being an at-risk drinker changed under the new guidelines.

Globally, alcohol causes approximately 5.9% of deaths each year and is linked to over 200 disease and injury conditions, accounting for 5.1% of the global burden of disease [5] and presenting a significant public health risk. In England in 2016, over 23,000 deaths were alcohol-related [6] and despite a gradual decrease in the reported alcohol consumption of men in England [7], the number of hospital admissions linked to alcohol consumption in England continues to rise [8]. In addition to health consequences for the drinker, alcohol is linked to numerous other consequences such as violence and crime [9, 10], child abuse and neglect [11, 12], reduced productivity and unemployment [13, 14] and many more intangible consequences such as reduced quality of life, pain and suffering, both for the drinker and those around them [15].

In response to concerns over alcohol-related deaths and illnesses, the first UK guidelines on individual drinking were produced in 1984 in *That’s the Limit*, with ‘safe limits’ described as 18 standard drinks (equivalent to 18 units) for men and 9 standard drinks for women [16]. Alcohol units (where 1 unit = 8 g pure alcohol) were introduced three years later along with a ‘sensible limits’ guideline of 21 units per week for men and 14 units per week for women, with ‘too much’ defined as 36 and 22 units respectively [16]. These guidelines continued until 1995, when, despite the British Medical Association [17] and the Royal Colleges of Physicians, Psychiatrists and General Practitioners [18] endorsing the continuation of the 21/14 units per week guideline, the *Sensible Drinking* guidelines changed to not regularly drinking more than 3 to 4 units per day for men and 2 to 3 units per day for women [19]. Whilst the move to daily limits caused concern amongst critics who viewed the change as, at best, being irrelevant and therefore ignored by the majority of drinkers who drink up to twice per week [20, 21] and at worst, encouraging daily drinking [22], all whilst significantly increasing the weekly limit [23]; in reality, the 21/14 units per week message has persisted alongside the daily guideline. This was demonstrated following the introduction of the latest guidelines in 2016, recommending that men and women drink no more than 14 units per week [3], when numerous headlines, including one from the Department of Health [24] reported a reduction in the weekly guidelines for men from 21 to 14 units [25, 26].

The latest guideline review was prompted by new evidence (since the 1995 guidelines) regarding the health risks associated with alcohol consumption, particularly related to cancer risks, where the risk of developing certain cancers (including, but not limited to cancers of the throat, mouth and breast) is now known to increase even at low levels of alcohol consumption [20]. The new guidelines were developed following a consultation which reviewed the recent evidence [27] and used the Sheffield Alcohol Policy Model to review morbidity and mortality risk estimates related to different alcohol consumption levels [28]. The resulting *Low Risk Drinking Guidelines* state that, for both men and women: “To keep health risks from alcohol to a low level it is safest not to drink more than 14 units per week on a regular basis” (3, p4) with additional guidance around spreading these units across three or more days. The decision to align the male and female weekly guideline was taken following evidence that at lower levels of alcohol consumption, the risks for men and women are similar, with men being more vulnerable to shorter-term risks and women more vulnerable to longer-term risks in general [3].

The aim of drinking guidelines is to inform the public in order for people to make informed decisions about their alcohol consumption [3, 29]. Whilst drinking guidelines have been criticised for their ineffectiveness in reducing alcohol consumption or changing drinking behaviour [30, 31], the value of drinking guidelines as part of a suite of education and information interventions, which also include health warnings on alcohol containers, counter-advertising and school interventions, has been recognised [1, 30]. Research into public awareness of changes to alcohol guidelines in the UK has repeatedly found that awareness peaks around the time of the change but is not sustained and long-term promotional campaigns have been recommended [32–34].

In addition to informing the public, drinking guidelines enable healthcare practitioners to assess and advise patients on their alcohol consumption. Screening for at-risk drinkers (usually either defined as drinking above national guidelines or identified via alcohol risk screening tools such as the Alcohol Use Disorders Identification Toolkit (AUDIT) [35]) and offering brief interventions for alcohol use, such as brief structured advice or brief motivational interviewing [36] have demonstrated positive results [37]. Whilst statistical modelling to predict population-level benefits of screening and brief interventions support universal screening [36], National Institute for Health and Care Excellence (NICE) guidance recommends universal screening if feasible and targeted screening of groups at greater risk of alcohol-related harm (e.g. people with relevant physical or mental health problems) if universal screening is not feasible [4]. Widespread implementation of screening has proved problematic both in trials of screening and brief intervention [38] and in clinical practice [39], and policy in England has favoured targeted screening in general practice settings, including the incentivisation of primary care services in England between 2008 and 2015 to deliver ‘Identification and Brief Advice’ for new patients only [36].

With an increasing focus within healthcare on targeting at-risk drinkers who consume above the low risk drinking guidelines [4, 37–39], the recent reduction in the low risk drinking threshold for men from 21 to 14 units per week could have a significant impact on healthcare providers as the number of at-risk drinkers increases. Furthermore, new awareness of the health risks associated with consuming above 14 units of alcohol per week means that supporting people to reduce their consumption to within these levels is a public health priority. Establishing the number of additional men who would be classified as at-risk following the guideline change may assist policy and commissioning decisions relating to screening for at-risk drinkers and provide a further incentive for improving education and awareness around drinking guidelines. Furthermore, within the English policy model of targeted screening for at-risk drinkers, it would be of benefit to know which demographic characteristics are associated with being an at-risk drinker and to establish whether the change in guidelines affected any specific demographic groups. It is feasible that men who were previously adhering to the guidelines may a) be more amenable to adhering to recommended guidelines having previously consumed within the low risk guidelines and b) not be aware that they are no longer considered to be drinking at low risk levels; therefore this group may benefit from specific targeting. Consequently, the objectives of this study were:

1. To establish the number of additional male at-risk drinkers in England over the past 5 years according to the new *Low Risk Drinking Guidelines*.
2. To establish which demographic characteristics in men were associated with being at-risk (> 21 units/week) vs low risk (≤21 units/week) drinkers according to the previous guidelines; which characteristics were associated with being at-risk (> 14 units/week) vs low risk (≤14 units/week) drinkers according to the new (2016) guidelines, and to explore whether the characteristics associated with being an at-risk drinker were different following the change to guidelines.

Whilst it could be argued that, in line with the 1995 *Sensible Drinking* guidelines of no more than 3 to 4 units per day for men [19], the change to guidelines constituted a 50% decrease from (a potential) 28 units per week to 14; both the media (e.g. 22–24), and the medical community [17, 18, 23, 40], including those defining increasing risk drinking in order to facilitate brief interventions in healthcare settings [4] viewed this as a reduction of one third in light of the pre-existing weekly limit of 21 units for men. Therefore, this paper will focus on a change in the *Low Risk Drinking Guidelines* from 21 units per week to 14 units per week for men.

## Methods

The Health Survey for England (HSE) is an annual, nationally representative, cross-sectional survey using a clustered, stratified multi-stage sampling design to select a random sample of private households in England. The survey is administered face to face within the participant’s household by a trained interviewer. Full details of the HSE methodology can be found online [41]. Ethical approval was sought prior to data collection from the relevant research ethics committee and further ethical approval was not required for this study.

### Measures

Questions on alcohol consumption have been included in the HSE since it began in 1991. Participants from 2011 to 2015 were asked if they ever drink: ‘*Do you ever drink alcohol nowadays, including drinks you brew or make at home?*’ and for those participants who respond no, a follow up question was asked: ‘*Could I just check, does that mean you never have an alcoholic drink nowadays, or do you have an alcoholic drink very occasionally, perhaps for medicinal purposes or on special occasions like Christmas and New Year?*’. For those who responded that they did drink alcohol, participants were asked about each type of alcohol in turn (e.g. normal strength beer, lager, stout, cider or shandy under 6% ABV; wine, including Babycham and champagne etc.) and were shown a card which demonstrates the types of drink and the different sizes of measure. Participants were asked how often they had consumed that beverage during the past 12 months (*1. Almost every day, 2. Five or six days per week…. 7. Once or twice a year, 8. Not at all in the last 12 months)* and then asked how much of that alcoholic drink they consumed on an average day when they were drinking it (one question for the size of measure and one for the number of drinks consumed). This information was then combined to calculate an average weekly consumption (the ‘quantity-frequency’ method) measured in units. Whilst quantity-frequency methods for collecting data on alcohol are common, the specific questions used in the HSE have been developed over time for this survey. The full HSE questionnaire (2015) can be accessed online [42] with the alcohol questions contained on pages 61 to 70 and show cards on pages 156 and 157. Further details on the background to the alcohol questions and how they have developed over time since the HSE began in 1991 can also be found online [43].

#### Objective 1

Using the average weekly alcohol consumption measure, men were regrouped into: 1) non-drinkers (no alcohol consumed in past 12 months); 2) low risk drinkers (≤14 units per week); 3) newly defined at-risk drinkers (> 14 to ≤21 units per week) and 4) previously defined at-risk drinkers (> 21 units per week).

#### Objective 2

Using the average weekly consumption measure, men were regrouped into:

1. At-risk (> 21 units per week) vs low risk (≤21 units per week) male drinkers according to the previous guidelines, and.
2. At-risk (> 14 units per week) vs low risk (≤14 units per week) male drinkers according to the new (2016) guidelines.

A number of demographic characteristics were identified from the literature as potentially impacting upon alcohol consumption levels and the corresponding measures from the HSE were selected for the analysis to establish whether at-risk male drinkers were distinct from low risk male drinkers under both the previous and the new drinking guidelines and to establish whether the change in guidelines affected the demographic characteristics associated with being an at-risk drinker. Demographic characteristics that have been associated with differing levels of alcohol consumption and were included in the analysis are: age [43], grouped from 16 to 34, 35–54, 55–74 and 75+; social class [44, 45], grouped using the National Statistics Socio-Economic Classification (NS-SEC) categorising the employment status of the participant (managerial and professional, intermediate, routine and manual, not classified); marital status [46, 47] grouped as single, married/cohabiting, separated/divorced/widowed; geographical region [6, 48–50], grouped by former Government Office Region; ethnicity [51, 52], regrouped into white and non-white groups due to small sample sizes in the non-white groups; smoking status [53], grouped by never smoker, ex-occasional smoker, ex-regular smoker, current smoker; and physical health [54, 55] measured as limiting long-lasting illness, non-limiting long-lasting illness, no long-lasting illness. The full HSE questionnaire (2015) can be accessed online [42].

### Sample

#### Objective 1

Data were utilized from all adult males (aged 16 and over), including non-drinkers and drinkers, but excluding those participants who had missing data on the alcohol measure (*N* = 121 (2011), *N* = 85 (2012), *N* = 135 (2013), *N* = 101 (2014), *N* = 223 (2015)). Data for the years 2011 to 2015 were selected because 2011 is the first year that the quantity-frequency measure was reinstated in the survey and 2015 was the most recent available dataset at the time that the data were analysed. Annual data were analysed separately. Sample sizes can be found in Table 1.

**Table 1:** Health Survey for England 2011–2015 Sample Sizes: Men aged 16+

Year	Non-drinkers/No alcohol in the past 12 months			Low Risk (≤14 units per week)			Newly Defined At-Risk (> 14 to ≤21 units per week)			Previously Defined At-Risk (> 21 units per week)			Total	
	Unweighted *N*	Weighted *N*	%	Unweighted N	Weighted *N*	%	Unweighted *N*	Weighted N	%	Unweighted *N*	Weighted *N*	%	Unweighted *N*	Weighted *N*
2011	454	515	12.8	1936	2123	52.7	406	451	11.2	905	941	23.3	3701	4030
2012	507	578	14.6	1829	2017	51.0	402	426	10.8	857	933	23.6	3595	3954
2013	553	611	14.7	1963	2151	51.9	393	447	10.8	881	940	22.7	3790	4150
2014	486	574	14.9	1873	2037	53.0	355	394	10.3	773	840	21.8	3487	3845
2015	483	525	13.7	1907	2104	55.0	391	421	11.0	722	776	20.3	3503	3825

#### Objective 2

Data from all adult (aged 16 and over) male participants from HSE 2015 who reported any alcohol consumption in the past 12 months were used, excluding those participants with missing data for the alcohol variable (*N* = 223). Participants with missing data from the other variables in the model were also excluded in the final sample. In all variables, the number of missing values constituted equal to or less than 1% of the sample (age: *N* = 0; NS-SEC: *N* = 31 (1.0%); marital status: *N* = 1 (< 1%); cigarette smoking status: *N* = 0; limiting long-lasting illness: *N* = 4 (< 1%); ethnicity: *N* = 2 (< 1%); former Government Office Region: *N* = 0). The final sample for each logistic regression model was 3258 with weighting applied (*N* = 2982 unweighted). Sample characteristics can be found in Table 2. The data used in the analysis are from 2015 (the most recent year of data at the time of the analysis). The focus of this paper was to estimate the number of men who would have been redefined as at-risk drinkers following the guidelines change and to explore the characteristics of at-risk drinkers under the previous vs the new guidelines, rather than to explore any effect of the change in guidelines itself; therefore using retrospective data was considered appropriate.

**Table 2:** Sample characteristics of low risk vs at-risk male drinkers under the previous guidelines (≤21 vs > 21 units/wk) and low risk vs at-risk male drinkers under the new guidelines (≤14 vs > 14 units/wk): HSE 2015

	Low risk drinkers (previous guidelines) *Previously defined low risk drinkers* ≤21 units per week			At-risk drinkers (previous guidelines) *Previously defined at-risk drinkers* > 21 units per week			Low risk drinkers (new guidelines) *Newly defined low risk drinkers* ≤14 units per week			At-risk drinkers (new guidelines) *Newly defined at-risk drinkers* > 14 units per week					
	Unweighted *N*	Weighted *N*	%	Unweighted N	Weighted N	%	Unweighted *N*	Weighted *N*	%	Unweighted N	Weighted N	%	Total un-weighted	Total weighted	Increase in % at-risk
Age 16+ in 20-year age bands															
16–34	504	776	81	117	182	19	427	653	68	194	306	32	621	958	13
35–54	766	864	75	251	291	25	647	735	64	370	420	36	1017	1155	11
55–74	717	632	71	299	261	29	567	499	56	449	394	44	1016	893	15
75+	282	218	86	46	34	14	241	187	74	87	66	26	328	252	12
NS-SEC															
Managerial and Professional	927	987	74	330	350	26	746	788	59	511	549	41	1257	1337	15
Intermediate	458	488	77	139	149	23	389	415	65	208	222	35	597	637	12
Routine and Manual	833	928	78	240	264	22	702	793	67	371	399	33	1073	1192	11
Other	51	86	93	4	6	7	45	76	83	10	16	17	55	92	10
Marital Status															
Single	360	542	77	116	161	23	304	458	65	172	245	35	476	703	12
Married/cohabitee	1640	1716	76	512	356	24	1339	1412	63	813	839	37	2152	2252	13
Separated/Divorced/Widowed	269	231	76	85	72	24	239	203	67	115	101	33	354	304	9
Government Office Regions															
North East	152	104	70	64	44	30	116	80	54	100	67	46	216	148	16
North West	278	298	70	121	129	30	217	239	56	182	188	44	399	427	14
Yorkshire and the Humber	217	238	78	65	68	22	178	197	64	104	109	36	282	306	14
East Midlands	226	218	78	62	62	22	199	193	69	89	87	31	288	280	9
West Midlands	201	251	75	72	83	25	160	200	60	113	134	40	273	334	15
East of England	288	301	80	76	76	20	247	260	69	117	117	31	364	377	11
London	226	336	76	69	106	24	192	278	63	103	164	37	295	441	13
South East	432	462	79	116	123	21	371	401	69	177	184	31	548	386	10
South West	249	282	78	68	78	22	202	225	62	115	135	38	317	631	16
Ethnicity															
White	2090	2256	75	687	735	25	1724	1869	62	1053	1122	38	2777	2991	13
Black/Asian/Mixed/Other	179	234	87	26	34	13	158	204	76	47	63	24	205	267	11
Cigarette Smoking Status															
Never smoked	1108	1280	83	237	261	17	944	1101	71	401	440	29	1345	1541	12
Ex-Occasional Smoker	107	119	72	38	47	28	88	98	59	57	68	41	145	166	13
Ex-Regular Smoker	684	656	71	279	270	29	545	525	57	418	401	43	963	926	14
Current Smoker	370	434	70	159	191	30	305	349	56	224	276	44	529	624	14
Long Lasting Illness															
Limiting Long-Lasting Illness	478	457	76	146	146	24	400	384	64	224	219	36	624	603	12
Non-Limiting Long-Lasting Illness	448	451	76	144	145	24	360	366	61	232	230	39	592	596	15
No Long-Lasting Illness	1343	1581	77	423	478	23	1122	1323	64	644	737	36	1766	2059	13
Total Men	2269	2490	76	713	769	24	1882	2073	63	1100	1185	37	2982	3258	13

### Statistical analyses

All data were analysed using the complex survey data procedures in Stata 15.0 and using the weighting variables provided in HSE to account for the clustered and stratified sampling.

#### Objective 1

Annual population prevalence estimates for newly defined at-risk drinkers (men drinking > 14 to ≤21 units per week) were calculated for each age group, following the procedure described in the HSE technical annexe for calculating population estimates [56]. The total annual population prevalence was calculated by summing the age group totals.

#### Objective 2

Non-drinkers were removed from the data as the *Low Risk Drinking Guidelines* apply to drinkers only. Firstly, a logistic regression model was applied to assess which characteristics were significantly associated with men being previously defined at-risk drinkers vs low risk drinkers (reference group) (men drinking > 21 vs ≤ 21 units/week), then a second logistic regression model was applied to assess which characteristics were significantly associated with men being newly defined at-risk drinkers vs low risk drinkers (reference group) (men drinking > 14 vs ≤14 units/week). The two models were then compared. As this was an exploratory analysis, all variables were entered into the model simultaneously.

## Results

1. To establish the number of additional male at-risk drinkers in England over the past 5 years according to the new *Low Risk Drinking Guidelines*

Unweighted and weighted sample sizes are recorded in Table 1. Sample sizes were different in each year, ranging from 3487 to 3790 (unweighted). Whilst exact percentages varied from year to year, the newly defined at-risk group (men drinking > 14 to ≤21 units per week) was consistently the smallest group of men, followed by non-drinkers and previously defined at-risk drinkers (men drinking > 21 units per week), with low risk drinkers constituting the majority of the sample each year.

Table 3 shows the annual population prevalence estimates of adult men in England (2011–2015) drinking at newly defined at-risk levels (> 14 to ≤21 units per week) by age group. The youngest (16–25 years) and oldest (75+ years) age groups consistently had amongst the lowest proportions of men drinking at newly defined at-risk levels, whilst the proportion of men drinking at newly defined at-risk levels in other age groups varied annually. The highest proportion in any age group categorised as newly defined at-risk drinkers was amongst 55 to 64-year-old men in 2015 (15.3%).

**Table 3:** Population estimates of men aged 16+ in England drinking at newly defined at-risk levels (> 14 to ≤21 units per week) 2011–2015

	Population Estimate for men drinking at newly defined at-risk (> 14 to ≤21 units per week)				
Age group	2011	2012	2013	2014	2015
16–24	267,202 (8.4%)	295,766 (9.3%)	303,767 (9.6%)	214,567 (6.8%)	202,012 (6.4%)
25–34	451,190 (12.6%)	337,064 (9.3%)	452,814 (12.3%)	440,510 (11.9%)	498,080 (13.3%)
35–44	511,584 (13.9%)	406,468 (11.3%)	395,989 (11.1%)	384,803 (10.9%)	337,036 (9.5%)
45–54	385,854 (10.7%)	380,888 (10.3%)	395,096 (10.6%)	446,348 (11.8%)	395,929 (10.4%)
55–64	394,857 (13.0%)	424,711 (14.2%)	306,709 (10.3%)	302,726 (10.1%)	467,067 (15.3%)
65–74	184,154 (8.5%)	282,813 (12.3%)	265,744 (11.1%)	230,572 (9.4%)	289,205 (11.5%)
75+	128,055 (8.1%)	134,078 (8.2%)	136,706 (8.2%)	162,875 (9.3%)	176,655 (9.9%)
Total	2,322,896	2,261,788	2,256,825	2,182,401	2,365,984
% Male Population	11.2	10.8	10.6	10.2	11.0
% Total Population	5.5	5.3	5.2	5.0	5.4

The percentage of men aged 16 and over, living in private residences in England who were drinking at newly defined at-risk levels ranged from 11.2% (2,322,896, 5.5% total population) in 2011 to 10.2% (2,182,401 men, 5% of the total population) in 2014.

2. To establish which demographic characteristics in men were associated with being at-risk (> 21 units/week) vs low risk (≤ 21 units/week) drinkers according to the previous guidelines; which demographic characteristics were associated with being at-risk (> 14 units/week) vs low risk (≤ 14 units/week) drinkers according to the new (2016) guidelines, and to explore whether the characteristics associated with being an at-risk drinker were different following the change to guidelines.

Sample characteristics for low risk vs at-risk male drinkers under the previous guidelines (men drinking ≤21 vs > 21 units/week) and low risk vs at-risk male drinkers under the new guidelines (men drinking ≤14 vs > 14 units/week) taken from Health Survey for England 2015 data are shown in Table 2. In all age groups, a higher proportion of men were classified as at-risk drinkers under the new guidelines, in line with the lower weekly threshold. The difference in percentage of at-risk drinkers under the previous vs new guidelines was greatest for 55–74 year olds (29% at-risk under the previous guidelines and 44% at-risk under the new guidelines) followed by 16–34 year olds (19% under the previous guidelines and 32% under the new guidelines).

Within the 2015 HSE sample, the demographic groups with the greatest difference in proportion of at-risk drinkers under the previous vs new guidelines were men working in managerial or professional occupations (26% of men under the previous guidelines vs 41% under the new guidelines), married or cohabiting men (24% vs 37%), men living in the North East (30% vs 46%) and South West (22% vs 38%) of England, white men (25% vs 38%), men who were current (30% vs 44%) or ex-regular (29% vs 43%) smokers and men with non-limiting long-lasting illness (24 vs 39%). For all demographic variables, the groups with the highest percentage of at-risk drinkers remained similar under the new guidelines.

Table 4 shows the results of two logistic regression models: column 1 shows the odds ratios for at-risk vs low risk (reference category) drinkers under the previous guidelines (> 21 vs ≤21 units/week) and column 2 shows the odds ratios for at-risk vs low risk (reference category) drinkers under the new guidelines (> 14 vs ≤14 units/week). All results relate to data from the HSE 2015.

**Table 4:** Logistic Regression Models for at-risk vs low risk male drinkers under previous guidelines (> 21 vs ≤21 units per week) and at-risk vs low risk male drinkers under the new guidelines (> 14 vs ≤14 units per week) (HSE 2015)

	At-risk vs low risk under previous guidelines (> 21 vs ≤21 units per week) OR (95% CI)	At-risk vs low risk under new guidelines (> 14 vs ≤14 units per week) OR (95% CI)
Age 16+ in 20-year age bands		
16–34	1.00	1.00
35–54	1.42 (1.08–1.89)*	1.18 (0.92–1.51)
55–74	1.71 (1.25–2.34)**	1.63 (1.25–2.12)***
75+	0.61 (0.40–0.92)*	0.69 (0.48–0.98)*
NS SEC		
Routine and Manual	1.00	1.00
Managerial and Professional	1.46 (1.15–1.86)**	1.64 (1.34–2.00)***
Intermediate	1.16 (0.86–1.55)	1.14 (0.88–1.49)
Other	0.30 (0.09–0.94)*	0.48 (0.22–1.05)
Marital Status		
Married/Cohabitee	1.00	1.00
Single	1.41 (1.06–1.88)*	1.28 (0.99–1.67)
Divorced/Separated/Widowed	0.96 (0.72–1.27)	0.78 (0.60–1.02)
Government Office Regions		
South East	1.00	1.00
North East	1.75 (1.09–2.79)*	2.08 (1.38–3.13)***
North West	1.78 (1.26–2.51)**	1.91 (1.41–2.60)***
Yorkshire and the Humber	1.22 (0.78–1.91)	1.42 (0.97–2.08)
East Midlands	1.14 (0.81–1.60)	1.06 (0.76–1.48)
West Midlands	1.37 (0.92–2.05)	1.68 (1.17–2.42)**
East of England	1.04 (0.69–1.55)	1.09 (0.75–1.61)
London	1.43 (0.96–2.14)	1.53 (1.03–2.28)*
South West	1.04 (0.72–1.51)	1.35 (0.97–1.88)
Ethnicity		
White	1.00	1.00
Black/Asian/Mixed/Other	0.44 (0.25–0.77)**	0.53 (0.34–0.80)**
Cigarette Smoking Status		
Never smoked	1.00	1.00
Ex-Occasional Smoker	2.10 (1.36–3.25)**	1.85 (1.25–2.74)**
Ex-Regular Smoker	2.11 (1.67–2.67)***	2.01 (1.63–2.47)***
Current Cigarette Smoker	2.30 (1.72–3.08)***	2.26 (1.73–2.94)***
Long-Lasting Illness		
No Long-Lasting Illness	1.00	1.00
Limiting Long-Lasting Illness	1.01 (0.77–1.34)	0.98 (0.77–1.25)
Non-Limiting Long-Lasting Illness	0.99 (0.76–1.28)	1.05 (0.85–1.30)
Constant	0.10 (0.07–0.15)***	0.19 (0.14–0.27)***

*P < 0.05 **p < 0.01 ***p < 0.001

Most demographic characteristics which were significantly associated with at-risk drinking under the previous guidelines remained so under the new guidelines. Under both the previous (OR 1.71, 95%CI 1.25–2.34) and new (OR 1.63, 95%CI 1.25–2.12) guidelines, 55–74 year old men had significantly greater odds of being classified as at-risk (vs low risk) drinkers when compared to the youngest age group (16–34 year olds); the association was slightly attenuated by the lowered threshold under the new guidelines. Under both the previous (OR 0.61, 95% CI 0.40–0.92) and new (OR 0.69, 95% CI 0.48–0.98) guidelines, men over 75 years old had significantly lower odds of being at-risk (vs low risk) drinkers when compared to the youngest age group.

Under the previous guidelines, 35–54 year olds had significantly greater odds of being at-risk drinkers (vs low risk drinkers) compared to the youngest age group (OR 1.42, 95% CI 1.08–1.98); however, under the new guidelines, 35–54 year old men no longer had significantly greater odds of being categorised as at-risk drinkers than the younger age group (OR 1.18, 95% CI 0.92–1.51). This was also the case in the unadjusted regression model (OR 1.22, 95% CI 0.97–1.54).

Men working in managerial or professional occupations had significantly greater odds of being classified as at-risk (vs low risk) drinkers compared to men working in manual or routine occupations under both the previous (OR 1.46, 95% CI 1.15–1.86) and the new (OR 1.64, 95% CI 1.34–2.00) guidelines, with the magnitude of association increasing following the change to the guidelines.

Under the previous guidelines, single men had significantly greater odds of being at-risk (vs low risk) drinkers compared to men who were married/cohabiting (OR 1.41, 95% CI 1.06–1.88); however, under the new guidelines this association was no longer significant (OR 1.28, 95% CI 0.99–1.67). It was also the case within the unadjusted regression model that single men did not have greater odds of being at-risk drinkers than married/cohabiting men under the new guidelines (OR 0.90, 95% CI 0.71–1.13).

Under both the previous and new guidelines, men from the North East (previous: OR 1.75, 95% CI 1.09–2.79; new: OR 2.08, 95% CI 1.38–3.13) and North West (previous: OR 1.78, 95% CI 1.26–2.51; new: OR 1.91, 95% CI 1.41–2.60) of England had greater odds of being classified as at-risk (vs low risk) drinkers than men from the South East of England. Under the new (but not the previous) guidelines, men from the West Midlands (OR 1.68, 95% CI 1.17–2.42) and London (OR 1.53, 95% CI 1.03–2.28) had greater odds of being classified as at-risk drinkers than those in the South East.

For men in the West Midlands, this association was also significant in the unadjusted model (OR 1.46, 95% CI 1.02–2.08). For men living in London, the association was not significant in the unadjusted model (OR 1.29, 95% CI 0.87-1.91), and when all variables were entered in a stepped process, the association was significant only when ethnicity was also included in the model.

Under both the previous (OR 0.44, 95% CI 0.25–0.77) and new (OR 0.53, 95% CI 0.34–0.80) guidelines, non-white men had significantly lower odds of being at-risk drinkers than white men.

Current smokers (previous: OR 2.30, 95% CI 1.72–3.08; new: OR 2.26, 95% CI 1.73–2.94), ex-regular smokers (previous: OR 2.11, 95% CI 1.67–2.67; new: OR 2.01, 95% CI 1.63–2.47) and ex-occasional smokers (previous: OR 2.10, 95%CI 1.36–3.25; new: OR 1.85, 95% CI 1.25–2.74) all had significantly greater odds of being at-risk drinkers under both the previous and the new guidelines. The association was slightly attenuated under the new, lower guidelines.

Neither limiting, nor non-limiting long-lasting illness were significantly associated with being classified as an at-risk drinker, either under the previous or the new guidelines.

## Discussion

The reduction in the *Low Risk Drinking Guidelines* for men from 21 to 14 units per week meant that between 2011 and 2015 over two million men each year, who would previously have been defined as drinking at low risk levels, would now be defined as at-risk drinkers. These newly defined at-risk drinkers constituted an additional 11% of the adult male population in England.

As health care services increasingly focus on screening for at-risk drinkers and offering brief interventions in order to reduce their alcohol intake [4, 37–39], the sudden increase in the number of at-risk drinkers by an additional 2.3 million men (based on 2015 figures) caused by the change to alcohol guidelines could have a significant impact on services, all at a time when incentivisation for identification and brief advice in primary care has been withdrawn [39]. Policy within England favours targeted screening [36]; a decision which is likely to have been influenced by the significant cost and practical implications of universal screening and the decision by the UK National Screening Programme not to recommend screening for alcohol misuse nationally [57]. It is therefore unlikely under the current system that these men will be identified through routine screening, and, particularly in light of the current evidence relating to poor awareness of changes to guidelines in the UK [32–34], these men may well continue to drink at levels which pose a risk to their health.

Knowing which groups of men have greater odds of being at-risk drinkers, and particularly those groups which would have been redefined as at-risk drinkers following the change to guidelines, could allow for 1) more targeted screening for at-risk male drinkers, and 2) targeted and sustained educational programmes around drinking guidelines.

Under both the previous and the new drinking guidelines, men aged 55–74 had greater odds of being at-risk drinkers, which correlates with current evidence around ‘middle-aged men’ being the most at-risk in terms of their drinking [58] and reinforces the need for more targeted campaigns for this age group. This could also provide an age criterion with which to screen for at-risk drinkers. Under the previous, but not the new guidelines, men aged 35–54 had greater odds of being at-risk drinkers, a change which may be explained by the greater increase in the proportion of at-risk drinkers in the reference category (16–34 year olds, 13% increase in at-risk drinkers) compared to 35–54 year olds (11% increase in at-risk drinkers). Around a third of men in the younger age group would now be considered at-risk from their drinking and the youngest age group experienced the second largest increase in the number of people considered at-risk under the new guidelines, indicating that a considerable proportion of this age group were drinking between 14 and 21 units per week. With this in mind, it is important to ensure that any sustained promotional activity does not focus on one age group to the detriment of others as has been suggested with the former focus on problematic younger drinkers [59].

Further criteria for targeted screening and education might include white men and men working in managerial and professional occupations, who had consistently greater odds of being at-risk drinkers in line with existing evidence [45, 52]. Routes to delivery here might be through screening and education in workplaces. The limited sample size only permitted analysis of a binary ethnicity category and further investigation of at-risk drinking within non-white ethnic groups is warranted. Whilst being single was associated with being an at-risk drinker under the previous guidelines, this was no longer the case under the new guidelines. This may be accounted for by the greater increase in the percentage of at-risk drinkers in the reference category (married/cohabiting men: 13% increase in at-risk drinkers) compared to single men (12% increase in at-risk drinkers). Whilst marriage is often associated with reduced drinking levels among men [60, 61], this finding indicates that drinking habits of married men could be putting them at-risk. Whether the higher number of married/cohabiting men drinking between 14 and 21 units indicates an attempt to drink within (previous) guidelines would require further investigation.

Targeted screening and the offer of brief interventions for alcohol consumption have been implemented differently across the devolved nations. In Wales, clusters of health behaviours are targeted [39] and the finding that current and ex-smokers had more than twice higher odds of being at-risk drinkers under both the previous and new drinking guidelines (consistent with the literature on the common co-occurrence of smoking and alcohol consumption [62, 63]), indicates that England could benefit from a similar system. In Scotland, there has been a stronger focus on screening and intervention in line with a more severe alcohol problem [36], providing evidence of the benefits of tailoring services according to geographical location. The change in guidelines strengthened the association between living in the North East and North West and being an at-risk drinker and under the new guidelines only, living in the West Midlands, and London (when adjusted for ethnicity), were also significantly associated with being an at-risk drinker. Therefore more tailored service provision of screening and interventions for alcohol could be put in place in the North East, North West, West Midlands and London, where men have odds between one and a half and twice higher of being at-risk drinkers.

### Strengths, limitations and future research

HSE is a nationally representative survey with consistently implemented alcohol measures, although it should be noted that HSE uses self-reported alcohol consumption measures and these have been found to under-report consumption [64], therefore the estimates in this paper may be conservative.

The current study sought to use retrospective data to explore the characteristics of men who would be considered at-risk from their drinking under the previous guidelines vs those considered at-risk under the new guidelines and therefore data from one year were used. Future research might use similar variables to explore cross-sectional changes over time to cover the period before and after the change to the guidelines.

## Conclusions

The change to the *Low Risk Drinking Guidelines* would have resulted in more than 2 million additional male at-risk drinkers in England. Men aged 55–74, current or ex-smokers, men in managerial or professional occupations and white men had greater odds of being at-risk drinkers under the new and previous guidelines and these are the groups already known to be drinking the most, strengthening the case for targeted screening and education around alcohol consumption for known groups of at-risk men. In addition, this study found that men in the West Midlands and London had greater odds of being at-risk drinkers under the new guidelines, and 16–35 year olds and married men had a marked increase in the proportion of at-risk drinkers. These are all groups that have not previously been identified as at-risk drinkers, and may benefit from specific education around the new *Low Risk Drinking Guidelines*.

## Data Availability

The datasets analysed within the current study are available from the UK Data Service: https://discover.ukdataservice.ac.uk/series/?sn=2000021

## Abbreviations

HSE: Health Survey for England
NatCen: National Centre for Social Research
NICE: National Institute for Health and Care Excellence
NS-SEC: National Statistics Socio-Economic Classification
SES: Socio-Economic Status
